# Improved Campephiline detection: An experiment conducted with the Magellanic Woodpecker

**DOI:** 10.1002/ece3.5671

**Published:** 2019-09-27

**Authors:** Amy L. Wynia, Virginie Rolland, Jaime E. Jiménez

**Affiliations:** ^1^ Department of Biological Sciences and Advanced Environmental Research Institute University of North Texas Denton TX USA; ^2^ Department of Biological Sciences Arkansas State University Jonesboro AR USA; ^3^ Universidad de Magallanes Punta Arenas Chile

**Keywords:** *Campephilus*, detection technique, drumming device, southern Chile

## Abstract

1. Woodpeckers can be difficult to detect, as they are often cryptic, secretive, occurring in low densities, and wary of humans. Several methods exist to detect woodpeckers (e.g., playback surveys, passive point counts), yet no research has established which technique best detects these elusive picids. Thus, we designed an experiment to determine which of three methods best results in a detection of Magellanic Woodpeckers (*Campephilus magellanicus*), and if weather variables influence detection probability.
2. Mostly during austral summers 2015–2017, we (a) used a drumming device to simulate a double‐knock (i.e., territorial acoustical signal), (b) broadcasted a territorial call, and (c) passively listened (control) for Magellanic Woodpeckers. We conducted our experiment on Navarino Island, Chile, where the Magellanic Woodpecker is the sole picid.
3. The drumming device most effectively influenced the likelihood of a woodpecker detection. The odds of a woodpecker responding to a double‐knock were 2.14 times more likely than responding to either a call or control. Moreover, the odds of a woodpecker detection decreased by 42% as wind increased by one category and decreased by 40% for every additional month (i.e., October–March), which was expected because woodpeckers become less territorial as the breeding season progresses.
4. As *Campephilus* woodpeckers communicate via drums or double‐knocks, using a drumming device likely will be an effective technique to detect not only Magellanic Woodpeckers, but other woodpeckers within the *Campephilus* genus in Central and South America.

## Graphical abstract

We designed an experiment to determine which of three methods best results in a detection of Magellanic Woodpeckers (*Campephilus magellanicus*). The drumming device most effectively influenced the likelihood of a woodpecker detection. Using a drumming device likely will be an effective technique to detect not only Magellanic Woodpeckers, but other woodpeckers within the *Campephilus* genus in Central and South America.

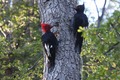

## 1 INTRODUCTION

Woodpeckers can be challenging to detect or locate (Kosinski & Kempa, 2007), as they are often secretive (Michalczuk & Michalczuk, 2006), quiet for long periods, overlooked among large trees that they inhabit (Allen & Kellogg, 1937), occupying wooded habitats with low visibility, cryptic (Kumar & Singh, 2010), or wary of humans (Conner, Jones, & Jones, 1994). Although some species or individuals may be more easily detectable by their drums or vocalizations (e.g., Drever, Aitken, Norris, & Martin, 2008; Vergara et al., 2017), not all woodpeckers express the same easily detectable behaviors or only may be more easily detectable seasonally. Additional factors such as small population sizes (Haig, Belthoff, & Allen, 1993), large home ranges (Tanner, 1964), low densities (Vergara et al., 2017), and steep and varying topography increase the difficulty of detecting woodpeckers. Therefore, passive methods (i.e., no detection device used) are often less reliable than active methods (i.e., use of detection devices). Accordingly, the best detection method may depend on several factors, including species‐specific behavior, habitat type, and season.

Various survey techniques have been used to estimate woodpecker abundances or densities. Such techniques include a variable‐belt‐width transect method (multiple species; Lammertink, 2004), playbacks of calls and drums with territory mapping (Black Woodpecker [*Dryocopus martius*]; Kosinski & Kempa, 2007, Pileated Woodpecker [*D. pileatus*]; Renken & Wiggers, 1993), only playbacks (Pileated Woodpecker; Drever et al., 2008), and passive point counts followed by an active survey method (multiple species; Kumar & Singh, 2010, Magellanic Woodpecker [*Campephilus magellanicus*]; Vergara et al., 2017). Despite various methods to detect and estimate woodpecker abundances or densities, to our knowledge, the most effective technique encompassing both calls and drums has never been reported.

**Figure 1:**
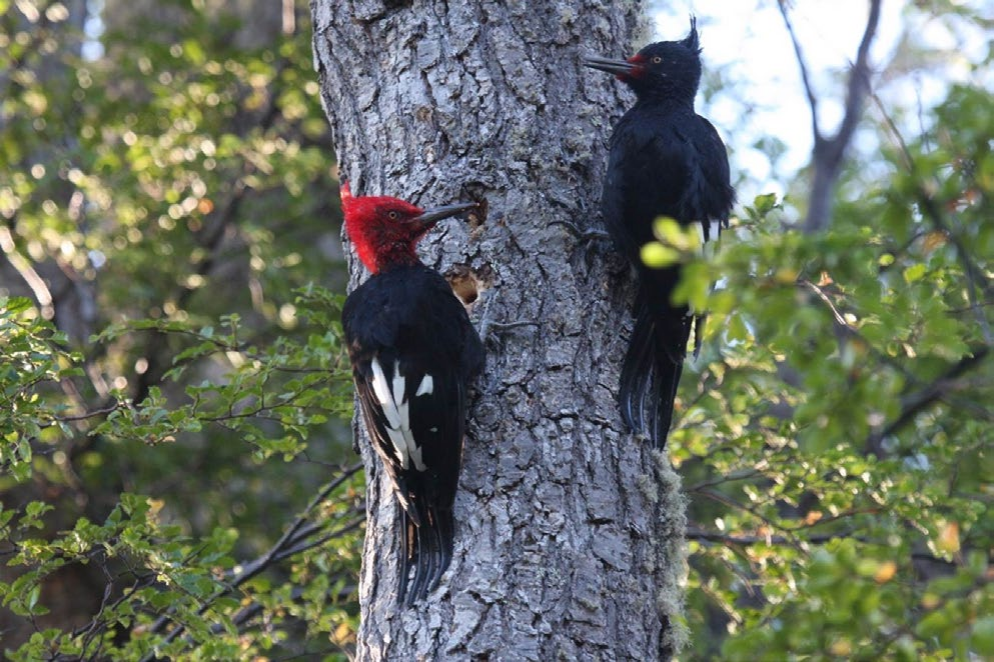
Male (left) and female (right) Magellanic Woodpeckers (*Campephilus magellanicus*) on Navarino Island, Chile. Photo by J. E. Jiménez

Related to the likely extinct Imperial (*C. imperialis*) and Ivory‐billed (*C. principalis*) woodpeckers, the Magellanic Woodpecker (hereafter MAWO) is currently the largest extant species of its genus and Central and South America (mean weight for males: 333 g [310–347 g, *n* = 27]; females: 303 g [240–340 g, *n* = 25]; A. L. Wynia, unpublished data). Males and females have black bodies with white wing patches; however, adult males are characterized by striking red head and neck plumage, whereas adult females have a long, black, curly crest, and red plumage near the base of their bills (Figure 1).

The *Campephilus* genus contains 12 large‐sized species (Winkler & Christie, 2002) that are native to the Americas. The Magellanic, however, is endemic to old‐growth forests of southern South America (Short, 1970) and is listed as endangered or vulnerable throughout its Chilean distribution (Servicio Agrícola y Ganadero [SAG], 2015).

MAWOs live in family groups of 2–5 individuals (Chazarreta, Ojeda, & Lammertink, 2012) with an average home range size of 1 km^2^ (Ojeda & Chazarreta, 2014). Particularly during the breeding season (i.e., mid‐late austral spring to early‐mid summer; Ojeda, 2004), MAWOs are highly territorial; disputes among family groups occur at home range boundaries or within territories (Soto et al., 2016). Disputes include aggressive behaviors such as chasing, double‐knocks, recognition calls (Soto et al., 2016), and supplanting (i.e., hopping/dancing‐like moves between woodpeckers on the same tree, A. L. Wynia, personal observation). Adult males are more aggressive, dominant (Chazarreta et al., 2012), and more frequently conduct a double‐knock (A. L. Wynia, personal observation), yet MAWOs generally travel with their family group (Ojeda, 2004); therefore, woodpecker families are often detected instead of individuals. Although this woodpecker is an important keystone species and of local conservation concern (Ojeda & Chazarreta, 2014), no standard technique has been established to detect and monitor populations.

Here, we address the following five questions: (a) Which of three detection methods (i.e., call, double‐knock, passive listening) is most effective in detecting MAWOs? (b) Which month is best to detect woodpeckers? (c) Does weather influence the likelihood of a woodpecker detection? (d) Does a specific detection method elicit a specific response type? (e) Does woodpecker detection time differ among detection methods?

Importantly, this study only accounts for detection probability (i.e., the likelihood of detecting a woodpecker using three different methods) without accounting for imperfect detection (e.g., Royle, Nichols, & Kéry, 2005). To account for this, researchers could deploy transmitters on a subset of woodpeckers and conduct the detection experiment with known woodpecker locations to determine their detectability; that is, given a woodpecker is present, does it respond to different detection techniques and at what distances?

**Figure 2:**
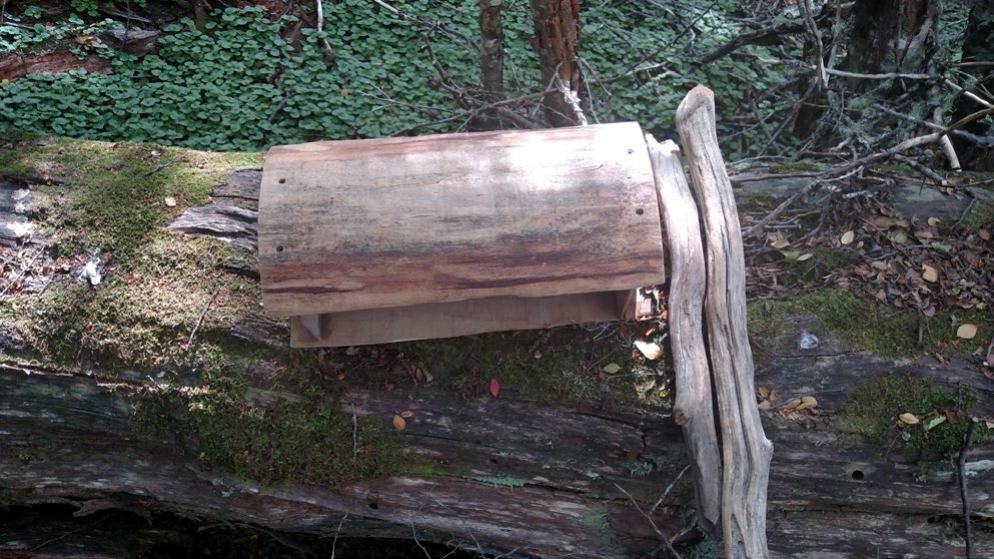
Wooden drumming device created to simulate a Magellanic Woodpecker (*Campephilus magellanicus*) double‐knock (i.e., territorial acoustical signal) on Navarino Island, Chile, 2015–2017

We designed an experiment to determine which of three detection methods would best elicit a MAWO detection. We predicted that the likelihood of a woodpecker detection would be higher with a drumming device (i.e., wooden, acoustical lure device used to simulate a double‐knock [i.e., territorial acoustical signal, Short, 1970]; Figure 2) than either a playback or passive listening, because drumming resonates louder and farther than playbacks, especially in windy environments (Vergara et al., 2017, A. L. Wynia, personal observation). Thus, we also predicted that wind would decrease the likelihood of a detection, because sound attenuates more rapidly in windy conditions. Importantly, we used this drumming device as opposed to broadcasting a recorded double‐knock with a speaker as the device could produce a louder sound that resonates more than anything broadcasted with our speaker (e.g., Vergara et al., 2017); this mimics the reality that a MAWO's double‐knock can be detected farther through a forest than its call.

Our main objective was to devise a detection technique that could then be used to estimate MAWO abundances or densities to better monitor population changes. Using active detection techniques to increase detection probability and estimate species abundances or densities is not uncommon (e.g., Jakob, Ponce‐Boutin, Besnard, & Eraud, 2010; Michalczuk & Michalczuk, 2006; Vergara et al., 2017). This research can provide valuable information for conservation and land managers that should assist in further protecting the MAWO, its habitat, and by association, co‐inhabitants as well. Also, our results likely can provide a detection technique applicable for other *Campephilus* species.

## 2 MATERIALS AND METHODS

### 2.1 Study site

**Figure 3:**
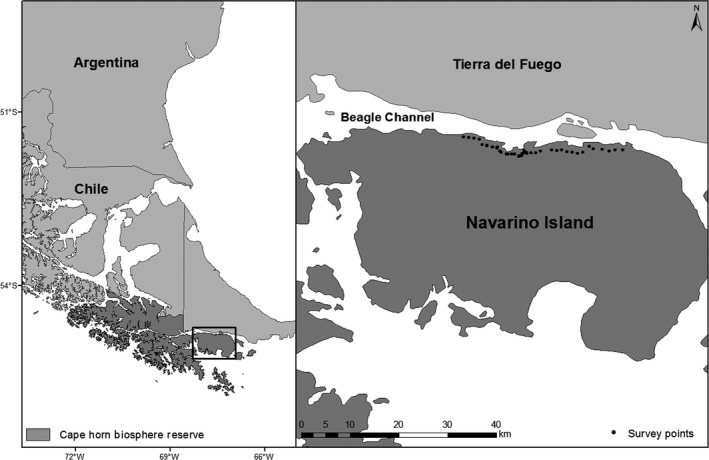
Navarino Island, Chile (55°04′S, 67°40′W), where detection methods for Magellanic Woodpeckers (*Campephilus magellanicus*) were compared in 2015–2017

The MAWO is a resident species of Navarino Island, Chile (55°04′S, 67°40′W; Figure 3), the location of this study. Navarino is 2,528 km^2^ (Lombardi, Cocozza, Lasserre, Tognetti, & Marchetti, 2010) and part of the Cape Horn Biosphere Reserve, which consists of an extensive archipelago in the Magellanic sub‐Antarctic ecoregion at the southern end of South America (Rozzi et al., 2012). Relatively harsh climatic conditions exist throughout the year, and of the few tree species inhabiting Navarino, several are *Nothofagus* (i.e., southern beech). Moreover, the MAWO is the only Picidae species inhabiting the island (Rozzi & Jiménez, 2014).

### 2.2 Methods

We conducted this experiment mostly during austral summers 2015–2017 (i.e., varying 3‐ or 4‐month periods between 12 October–12 March). During a pilot study in summer 2015 (i.e., 25 January–12 March), we established 12 forested survey points along the accessible, northern coast of Navarino; these points were sampled for one season and were included in the analyses. During austral summer 2015–2016, we established an additional 30 forested survey points that were resampled in 2016–2017. They were randomly selected between 50–500 m south of the only road to reduce road effect and were 1 km apart to reduce detecting the same woodpecker families repeatedly. We randomized all starting survey points during each survey period for every field season to prevent visiting the same location at the same time on every survey. At each point, we randomly chose one of three detection methods: (a) passively listened (control); (b) broadcasted a MAWO territorial call; or (c) simulated a double‐knock with a drumming device (Figure 2). Each detection method was used approximately once per point per field season (for three seasons) to reduce potential diminishing responsiveness in woodpeckers.

For the active techniques, we either played a short territorial call (http://www.xeno-canto.org: XC52601) via a speaker (Altec Lansing Mini H20 model IMW257) at about 55 dB for approximately 10 s or simulated a double‐knock with the drumming device. We did not measure the sound pressure level (dB) of the drumming device because it depended on multiple factors (e.g., substrate on which the device was placed, user's strength, location hit on the device). The device, created on Navarino from lenga wood (high deciduous southern beech, *N. pumilio*), had two 40‐cm × 19.5‐cm sides and two 9‐cm × 19.5‐cm sides that were inset by 7 cm on each long side (Figure 2). Two open sides projected the sound produced by the double‐knock that we created with sticks found in the forest. We repeated the active techniques three times (i.e., once about every 3.5 min) during a 10‐min period or passively listened for 10 min. Using a Kestrel 3000 Wind Meter, we recorded average wind speed (km/hr) and temperature (°C); we also recorded cloud cover (%), start time of each simulation, woodpecker behavioral response (e.g., call, double‐knock, visually approach, no response), detection time, and estimated distance from survey point (m) at first detection. We repeated this experiment 3–4 times (i.e., monthly) per field season between 04:45–15:30 local time, as woodpeckers are active and responsive throughout the day (Kumar & Singh, 2010; Vergara & Schlatter, 2004).

### 2.3 Analyses

We performed all statistical analyses with R statistical software version 3.5.0 (R Core Team, 2018). We set the significance level at 5% and reported 95% confidence intervals (CIs) or limits (CLs), and means with standard errors. If CIs included 0, predictors were not significant. We checked for outliers (there were none) and multicollinearity among predictors (package usdm; Naimi, Hamm, Groen, Skidmore, & Toxopeus, 2014). Our global model was not overdispersed ($\hat{c}$ = 0.99), nor was there multicollinearity among predictors (i.e., no variance inflation factor (VIF) value was >10). There was no effect of year (*p* = .79, CI = −0.42–0.44) on the probability of a woodpecker detection; therefore, all years were combined, and survey points remained the only random effect in our mixed models.

For question 1 on the best detection method, we used a generalized linear mixed model (GLMM, package lme4; Bates, Maechler, Bolker, & Walker, 2015) with a binomial error distribution. The data set included all woodpecker detections during each 10‐min survey. For questions 2 and 3 on month and weather effect, respectively, we used a GLMM with an offset to account for uneven detections per month and used the first detection during each survey. We considered the following variables: temperature, cloud cover, wind speed, detection method, month, and survey time. We created categories for all variables but month (Table 1); wind speed categories followed the Beaufort wind force scale (WMO, 1970). We created our a priori global model based on all independent environmental and temporal variables, detection method, and relevant interactions; we created all possible model combinations (package MuMIn, function dredge) and used an information‐theoretic approach with the Akaike Information Criterion corrected for small sample size (AICc; Burnham & Anderson, 2002) to select the best‐supported model. We applied the principle of parsimony if ΔAICc < 2. Additionally, to determine the magnitude of the effect of influential predictors, we computed odds ratios and reported 95% CLs. If CLs included 1, the predictor had no influence on the likelihood of a woodpecker detection.

**Table 1:** Categories created for survey time period, temperature (°C), and wind speed (km/hr) for 10‐min detection surveys for Magellanic Woodpeckers (*Campephilus magellanicus*) on Navarino Island, Chile, 2015–2017

Category	Time	Temperature (°C)	Wind (km/hr)
1	04:00–06:00	0.0–5.0	0.0–1.0
2	06:01–08:00	5.1–10.0	1.1–5.0
3	08:01–10:00	10.1–15.0	5.1–11.0
4	10:01–12:00	15.1–20.0	11.1–19.0
5	12:01–14:00	20.1–25.0	NA
6	14:01–16:00	NA	NA

Note

We used the Beaufort scale of wind force (WMO, 1970) for wind categories and used all categories for model selection.

For question 4 on response type, we used a multinomial logistic mixed‐effects model (MLMM; package lme4; Bates et al., 2015) with response types (i.e., call, double‐knock, other, and no) as response variables and used all detections during each survey. Finally, for question 5 on detection time, we used a mixed‐effect ANOVA (package stats; R Core Team, 2018) and used the first detection during each survey.

## 3 RESULTS

**Figure 4:**
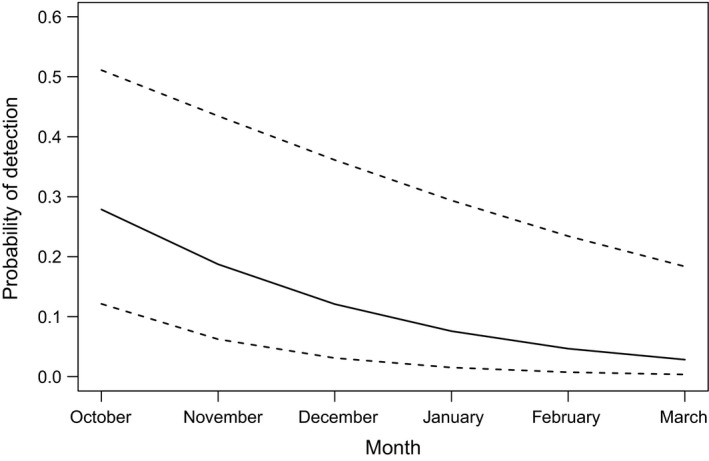
Probability (solid line) with 95% confidence intervals (dashed lines) of detecting Magellanic Woodpeckers (*Campephilus magellanicus*) monthly on Navarino Island, Chile, 2015–2017

The drumming device most effectively influenced the likelihood of a woodpecker detection (*p* = .02). The odds of a woodpecker responding to a double‐knock were 2.14 times more likely than responding to either a call or control (Table 2). In general, the number of detections per survey point varied between 0–5, and the type of woodpecker response and number of responses to each detection method varied as well (Table 3). A significant difference occurred in monthly woodpecker detections (*p* < .01); for each additional month (i.e., October–March), the odds of detecting a woodpecker (for all methods) decreased by 40% (Table 2). Thus, we were more likely to detect a woodpecker earlier in the breeding season than later (Figure 4).

**Table 2:** Parameter estimates with standard errors (*SE*) and odds ratio estimates with 95% confidence limits (CL) for the odds of detection method, wind, and month influencing the likelihood of a Magellanic Woodpecker (*Campephilus magellanicus*) detection during a detection survey on Navarino Island, Chile, 2015–2017

Parameter	Estimate ± *SE*	Odds ratio	
		Estimate	95% CL
Intercept (control)	−0.54 ± 0.35	0.58	0.30–1.15
Call	0.00 ± 0.31	1.00	0.55–1.84
Double‐knock	0.76 ± 0.31	2.14	1.16–3.96^a^
Intercept (of wind)	−0.26 ± 0.41	0.77	0.34–1.73
Wind	−0.55 ± 0.25	0.58	0.35–0.95^a^
Intercept (of month)	−0.43 ± 0.40	0.65	0.29–1.43
Month	−0.52 ± 0.11	0.60	0.48–0.74^a^

a Significant parameter (CL does not include 1).

**Table 3:** Survey point, number of Magellanic Woodpecker (*Campephilus magellanicus*) detections per point, detection method used that resulted in a detection, woodpecker response type, frequency of each response, and time (min) at first woodpecker detection during 10‐min detection surveys conducted on Navarino Island, Chile

Survey point	No. detections	Detection method	Response type	Response frequency	Detection time (min)
1	0	None	NA	NA	NA
2	2	Co, Ca	Ca, DK	4, 2	5, 1
3	1	DK	DK	7	1
4	2	Ca, Ca	DK, DK	8, 8	1, 6
5	4	DK, DK, Ca, Co	V, P, Ca, DK	2, 4, 5, 7	3, 3, 0, 1
6	3	DK, Ca, Co	DK, Ca, Ca	1, 7, 2	8, 1, 5
7	0	None	NA	NA	NA
8	0	None	NA	NA	NA
9	1	DK	Ca	1	0
10	1	DK	DK	2	5
11	3	Ca, DK, Ca	Ca, DK, DK	1, 4, 1	7, 0, 1
12	2	Ca, DK	Ca, DK	4, 3	8, 6
13	1	Ca	DK	2	1
14	2	Co, DK	Ca & DK, DK	2, 1	6, 9
15	0	None	NA	NA	NA
16	0	None	NA	NA	NA
17	3	Ca, Co, DK	Ca, Ca, DK	8, 1, 5	1, 3, 4
18	1	Co	P	2	0
19	5	Ca, DK, Co, DK, DK	V, DK, Ca, DK, Ca	3, 1, 8, 1, 7	3, 2, 8, 1, 4
20	2	DK, Co	P, DK	1, 2	3, 4
21	3	DK, Ca, Ca	DK, DK, DK	1, 3, 2	0, 3, 6
22	1	DK	Ca	2	5
23	2	DK, DK	DK, DK	5, 1	5, 2
24	3	Co, Co, Ca	Ca, Ca, F	8, 3, 1	1, 0, 8
25	2	DK, Co	DK, F	11, 2	3, 3
26	1	Co	Ca	1	0
27	0	None	NA	NA	NA
28	2	DK, DK	Ca, Ca	2, 2	2, 1
29	3	Co, Ca, Co	Ca, DK, DK	3, 2, 8	4, 3, 3
30	4	DK, Ca, DK, Co	Ca, Ca, DK, DK	5, 9, 3, 2	0, 8, 5, 2
31	1	Co	DK	1	10
32	1	Co	Ca	9	0
33	2	DK, Ca	DK, Ca	2	5
34	1	DK	Ca	1	8
35	0	None	NA	NA	NA
36	1	Ca	Ca	7	0
37	2	Co, DK	DK, DK	3, 5	4, 8
38	0	None	NA	NA	NA
39	1	DK	Ca	3	6
40	0	None	NA	NA	NA
41	0	None	NA	NA	NA
42	1	DK	Ca	13	9

Note

Time 0 min indicates a detection occurred within the first min of a survey. Survey points 1–30 were visited seven times across two austral field seasons (2015–2017), whereas points 31–42 were visited three times during one field season (2015). “None” implies no method resulted in a detection. The order listed per row in detection method corresponds to the order in remaining columns.

Abbreviations: Ca, call; Co, control; DK, double‐knock; F, flying (heard, not seen); NA, not applicable; P, pecking; V, visual.

**Figure 5:**
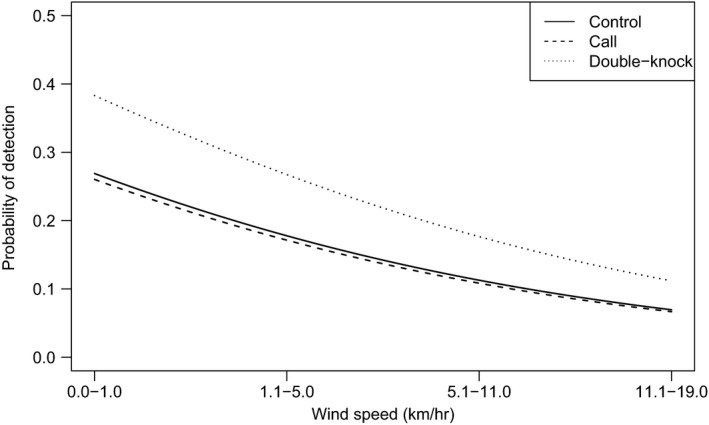
Probability of detecting Magellanic Woodpeckers (*Campephilus magellanicus*) using various detection methods relative to wind speed (km/hr) on Navarino Island, Chile, 2015–2017. Wind categories follow the Beaufort wind force scale (WMO, 1970)

The best‐supported models all included wind as a predictor of a woodpecker detection (Table 4), but the most parsimonious model contained wind only. Specifically, the odds of a woodpecker detection decreased by 42% as wind increased by one category (Table 2, Figure 5).

**Table 4:** Results of model selection for a priori models with ∆AICc < 2 containing potentially influential temporal and biological variables, detection method, or interaction effects that may influence the likelihood of a Magellanic Woodpecker (*Campephilus magellanicus*) detection during a detection survey on Navarino Island, Chile, 2015–2017

Candidate model	*K* a	AICc	∆AICc^b^	*ω_i_* c	LL^d^
Cloud + wind	4	278.48	0.00	0.17	−135.15
Wind^e^	3	278.48	0.00	0.17	−136.19
Method + wind	5	279.65	1.17	0.09	−134.70
Temperature + wind	4	279.74	1.27	0.09	−135.79
Month + wind	4	279.75	1.28	0.09	−135.79
Cloud + method + wind	6	279.77	1.29	0.09	−133.71
Cloud + temperature + wind	5	280.00	1.53	0.08	−134.88
Cloud + time + wind	5	280.01	1.54	0.08	−134.88
Time + wind	4	280.04	1.56	0.08	−135.94
Cloud + month + wind	5	280.05	1.57	0.08	−134.90

a Number of parameters.

b Difference in corrected Akaike's Information Criterion (∆AICc = AICc*_i_*‐min. AICc).

c Model weight (i.e., explanatory power).

d Log likelihood.

e The best‐supported model with fewest number of parameters.

Regardless of detection method used, there was no difference in woodpecker response type (e.g., call, double‐knock, visual, no response) between the control and call (*z* = 1.78, *p* = .08) or double‐knock methods (*z* = 1.49, *p* = .14), nor between the call and double‐knock methods (*z* = −0.28, *p* = .79). Finally, mean woodpecker detection time (i.e., at first detection) did not differ among detection methods (*F*_2,30_ = 0.18, *p* = .84). Mean detection times were 3.3 ± 0.67 min for the control, 3.3 ± 0.70 min for the call, and 3.9 ± 0.54 min for the drumming device.

## 4 DISCUSSION

Woodpecker drumming, that is, rapid, repetitive strikes with a bill on a substrate that is not associated with foraging or excavating, is used for long‐distance communication with conspecifics in mate selection and territoriality (Stark, Dodenhoff, & Johnson, 1998 and references therein). Given that double‐knocking is the main long‐distance territorial signal in *Campephilus* species (Short, 1970), we suggest researchers simulate double‐knocks to increase detection probability. We further recommend the use of a drumming device over broadcasting double‐knocks with a speaker, because (a) speakers may not broadcast at the same sound pressure level (dB) and (b) speakers that could broadcast loudly can be more expensive. The speaker used in our study (Altec Lansing Mini H20 model IMW257) costs $30 USD with a maximum sound pressure of 55 dB, whereas, for example, the speaker used by Castro, De Rosa, Priyadarshani, Bradbury, and Marsland (2019) (Saul Mineroff Portable Field Speaker model SME‐AFS) cost $295 USD with a maximum sound pressure of 105 dB (Saul Mineroff Electronics, Inc.). Including parts and labor, the cost of the drumming device is approximately $10 USD; this device can produce a loud sound (although its sound pressure level was not measured) and is more cost‐effective than a speaker.

Another benefit to the drumming device is its simplicity; it never needs to be charged, it will not die in the field, and batteries do not need to be replaced. Moreover, modifications to our drumming device to increase woodpecker detections may better assist researchers in detecting and monitoring woodpecker populations in the *Campephilus* genus. Modifications could include adjusting device dimensions, using different wood or drumming sticks, or training researchers to increase accuracy of drum mimicry. At a minimum, the drumming device should help establish baseline presence/absence data or contribute to occupancy modeling.

In this study, we report that MAWOs were 2.14 times more likely to respond when we used a drumming device. Similarly, Kumar and Singh (2010) reported that individuals of 11 woodpecker species in India were 2.2 times more likely to respond during a playback survey than during a visual/aural survey. However, they only broadcasted calls, not drums; therefore, the impact of drumming is unknown. Furthermore, woodpecker territoriality was not discussed, which may have impacted their results.

MAWOs are highly territorial against conspecifics during the breeding season; therefore, an active detection technique (i.e., a drumming device) can elicit a detection more readily than passive techniques. MAWOs often approach the “intruder” (i.e., playback or drumming device‐ at times within meters, A. L. Wynia, personal observation) and are generally tolerant of humans (Ojeda & Chazarreta, 2014); therefore, researchers likely can increase the success rate of capture attempts and better identify individuals and observe their behaviors at close range to address other research questions. Examples include identifying banded individuals, estimating woodpecker family sizes, or observing family or territorial interactions.

Because MAWOs are territorial, we used a territorial call to increase detection probability; however, this less likely influences juveniles or nonterritorial individuals. Only one vocalization type was used to reduce potential vocalization effect; thus, varying the call type may have impacted the detectability of individuals. For example, playing a juvenile begging call increases the likelihood of detecting females, as females more readily respond by approaching the sound (A. L. Wynia, personal observation during capture attempts). However, of all known detections by sex, observing only females responding to the territorial playback occurred less frequently (i.e., 20.69%; *n* = 6/29), whereas observing just males (27.59%; *n* = 8/29) or both sexes (51.72%; *n* = 15/29) occurred more frequently. Notably, of all known detections by sex for all methods (*n* = 47), only 14.89% (*n* = 7/47) of detections came from solo females. Therefore, further research should address the importance of using various vocalizations for active detection techniques.

Previous studies have reported increased woodpecker detections with use of playbacks (e.g., Kumar & Singh, 2010; Michalczuk & Michalczuk, 2006), but surprisingly, there was no difference between the playback and control in our study. Perhaps if we had used a different vocalization, we may have increased our detection probability. However, given the large home range size of MAWOs (i.e., 1 km^2^), it is likely woodpeckers were not within hearing distance of the quieter playback. The windy environment of Navarino likely attenuated the playback as well, which limited the distance at which a woodpecker could detect it. Although our speaker likely did not transmit the playback as far as a true MAWO territorial call, the lower amplitude of the playback relative to the simulated double‐knock imitates the reality that MAWO calls do not carry as far through the forest as their double‐knocks. However, as we did not compare the sound pressure level (dB) of a natural MAWO call nor the simulated double‐knock, we cannot exclude the possibility that MAWOs were detected more frequently with the drumming device than the broadcasted call simply because the sound broadcasted farther in the forest. Yet, double‐knocking is the MAWOs primary long‐distance territorial signal; this suggests that double‐knocking would increase a MAWO's detection probability, regardless of the sound pressure level. To test this, researchers could broadcast a double‐knock and call with a speaker to determine which method increases detection probability.

Regardless of detection method, the probability of detecting a woodpecker significantly decreased monthly (i.e., October‐March). As the austral breeding season progresses, woodpeckers become less territorial (G. E. Soto, unpublished data) and less responsive to active detection techniques. Thus, greater response frequency earlier in the breeding season suggests MAWOs were defending their territories more intensively than later in the season. Previous research on Pileated Woodpeckers, a similar‐sized North American species, suggests vocalizations and drumming decline throughout the breeding season (Tremain, Swiston, & Mennill, 2008). Therefore, we suggest researchers should conduct active survey methods earlier in the breeding season to maximize woodpecker detections. Importantly, our study was mainly conducted during the breeding season; therefore, detection probability in response to active techniques in nonbreeding seasons remains unknown but is likely reduced.

Independent of month, wind varied the distance at which the drumming device or playback could be heard. In other regions of Chile, with minimal wind, the drumming device was heard up to 300 m from the bottom of a steep valley with dense forest, and as far as 2.5 km from a steep ridgetop (G. E. Soto, personal communication 31 August 2018). As we conducted this study at lower elevation in varying habitats of dense or old‐growth forests often with forest edges adjacent to beaver meadows and with varying degrees of wind, our double‐knocks were likely detected up to 300 m and calls up to 75 m. In windy and forested environments, the higher frequency of a woodpecker playback is attenuated much faster than the lower frequency of a double‐knock.

Especially in windy environments, sounds and vocalizations may go unheard or are abruptly dampened. Even without wind, some sounds are still difficult to detect. For example, the sound of MAWOs foraging is not very loud (Short, 1970), as is common with other woodpecker species (Lammertink, 2004). Their wings, however, produce a flapping sound (Short, 1970), which can be detected and uniquely identified particularly on Navarino (as it is the only picid) in a relatively quiet environment. If audible, these nuances may increase detectability for seasoned researchers, but likely will be missed by untrained or inexperienced investigators. Moreover, a drumming device additionally could assist novice or inexperienced researchers, as less skill is required to identify the species by call or drum because woodpeckers often respond or move toward the observer (Kumar & Singh, 2010). Therefore, using a drumming device should increase the likelihood of detecting otherwise quiet woodpeckers for all researchers.

Although the drumming device increased detection probability, there was no difference in woodpecker response type (i.e., call, double‐knock, or other) per detection method. Of our 426 surveys, 244 (57.3%) resulted in a response, 182 (42.7%) yielded no response. This, however, does not necessarily imply that woodpeckers were absent. Perhaps (a) woodpeckers were not within hearing distance of the active technique, (b) incubating/brooding females may not wish to disclose their location to rival conspecifics (Tremain et al., 2008), or (c) transient or less‐dominant individuals may choose to remain undetected. Moreover, woodpeckers may not have responded during the control as there was no potential “threat” to their territory.

Similar to response type, mean detection time did not differ among methods, although detection time was slightly longer for the double‐knock. Given that a double‐knock can be detected farther than a call, we speculate that Magellanic Woodpeckers were farther away in their territory when they detected the simulated double‐knock and took longer to respond. Indeed, 85.7% (*n* = 24/28) of responses to the drumming device were recorded ≥50 m from the survey point, whereas only 61.1% (*n* = 11/18) of responses to a playback were recorded ≥50 m from the survey point. Yet, Kumar and Singh (2010) reported that woodpeckers were detected faster during the playback survey than during the visual/aural survey; 45% (*n* = 111) and 83% (*n* = 204) of individuals responded within 15 s and 60 s, respectively. In our study, mean MAWO responses were 3–4 min for all methods. Notably, Kumar and Singh (2010) did not record detection distance. Perhaps factors such as territory size, territoriality, wind speed, or forest structure influence the difference in woodpecker detections between the sub‐Himalayan tropical/subtropical forests of India and the temperate forest of southern Chile.

Specifically for MAWOs, we suggest researchers use a drumming device earlier in the breeding season (i.e., October and November) on days with low wind to increase detection probability. Survey points should be approximately 1 km apart to reduce detecting identical families and inflating estimates. As woodpeckers are responsive throughout the day, survey time is less important; however, we recommend starting earlier in the morning (i.e., close to sunrise) to increase the amount of time available to conduct surveys. Importantly, we only simulated a double‐knock once every 3.5 min during the detection survey to standardize the number of simulations between active techniques. MAWOs generally conduct double‐knocks more rapidly (i.e., between 30–120 s apart [e.g., https://macaulaylibrary.org: *Campephilus magellanicus* (ML235915)]). Therefore, researchers could increase the frequency of double‐knocks to likely increase either the speed or frequency of detection. Finally, one may consider the use of loudspeakers if those can reproduce the woodpecker double‐knock at the natural sound pressure level.

Detecting and monitoring woodpecker populations is particularly important as several species are declining or endangered (Mikusińki, 2006). Notably, little research has been conducted on the *Campephilus* genus (Ojeda, 2004). Our study suggests that a drumming device is an effective alternative to playbacks to establish baseline population estimates, a primary conservation objective for all *Campephilus* species, including MAWOs.

## CONFLICT OF INTEREST

None declared.

## AUTHOR CONTRIBUTIONS

ALW and JEJ conceived the ideas and designed the methodology, ALW collected the data, ALW and VR analyzed the data, and ALW led the writing of the manuscript. All authors contributed critically to the drafts and gave final approval for publication.

## Data Availability

Original data and analyses pertaining to this research are available on the Dryad Digital Repository: https://doi.org/10.5061/dryad.78dj7t9.
